# An efficient algorithm for de novo predictions of biochemical pathways between chemical compounds

**DOI:** 10.1186/1471-2105-13-S17-S8

**Published:** 2012-12-07

**Authors:** Masaomi Nakamura, Tsuyoshi Hachiya, Yutaka Saito, Kengo Sato, Yasubumi Sakakibara

**Affiliations:** 1 Biosciences and Informatics, Keio University, 3-14-1 Hiyoshi, Yokohama 223-8522, Japan

## Abstract

**Background:**

Prediction of biochemical (metabolic) pathways has a wide range of applications, including the optimization of drug candidates, and the elucidation of toxicity mechanisms. Recently, several methods have been developed for pathway prediction to derive a goal compound from a start compound. However, these methods require high computational costs, and cannot perform comprehensive prediction of novel metabolic pathways. Our aim of this study is to develop a *de novo *prediction method for reconstructions of metabolic pathways and predictions of unknown biosynthetic pathways in the sense that it does not require any initial network such as KEGG metabolic network to be explored.

**Results:**

We formulated pathway prediction between a start compound and a goal compound as the shortest path search problem in terms of the number of enzyme reactions applied. We propose an efficient search method based on A* algorithm and heuristic techniques utilizing Linear Programming (LP) solution for estimation of the distance to the goal. First, a chemical compound is represented by a feature vector which counts frequencies of substructure occurrences in the structural formula. Second, an enzyme reaction is represented as an operator vector by detecting the structural changes to compounds before and after the reaction. By defining compound vectors as nodes and operator vectors as edges, prediction of the reaction pathway is reduced to the shortest path search problem in the vector space. In experiments on the DDT degradation pathway, we verify that the shortest paths predicted by our method are biologically correct pathways registered in the KEGG database. The results also demonstrate that the LP heuristics can achieve significant reduction in computation time. Furthermore, we apply our method to a secondary metabolite pathway of plant origin, and successfully find a novel biochemical pathway which cannot be predicted by the existing method. For the reconstruction of a known biochemical pathway, our method is over 40 times as fast as the existing method.

**Conclusions:**

Our method enables fast and accurate *de novo *pathway predictions and novel pathway detection.

## Background

Identification of the metabolic pathway of a chemical compound and discovery of new metabolic pathways are important in various fields. In general, an enzyme reaction pathway is a sequence of applications of enzymes (represented by EC number) that derives a goal compound from a given compound. In the field of drug discovery [1], the mechanism of side effects based on information about metabolic pathways has been investigated to clarify the movement of drugs in the body and to optimize drug candidate compounds. In the field of toxicity prediction, exploration of the dynamics of *in vivo *chemical substances identified the metabolic pathway, leading to the elucidation of the mechanisms of toxicity [2]. Prediction of the biochemical pathways for secondary metabolites has received the most attention in recent years. Although secondary metabolites have been used as lead compounds for food and medicines, most of their biosynthetic pathways still remain unknown. Further, computational methods that support *de novo *design of biosynthetic pathways are expected in the field of synthetic biology. In the synthetic biology approach, the *de novo *design is not necessarily limited to biochemical routes that already exist in nature [3].

To solve the problem of predicting various metabolic pathways, many attempts from bioinformatics have been made so far. Existing approaches can be broadly divided into three methods: the fingerprint-based method, the maximum common substructure search method, and the reaction rule-based method.

### Fingerprint-based method [4]

A chemical compound is represented by a fingerprint of the molecular structure, and the Tanimoto coefficient between fingerprints for compounds is calculated to indicate similarity. It then predicts that there is a metabolic pathway between compounds if the similarity exceeds a certain threshold. The necessary calculations are fast, but accurate path prediction is difficult.

### Maximum common substructure search method [5,6]

This approach focuses on the maximum common substructure between compounds to predict a metabolic pathway. The maximum common substructure search is an NP-hard problem, and requires enormous computation time in order to evaluate the similarity between compounds of complex structures [7,8]. Various approximation algorithms have been studied in the search for a computationally tractable approach [9-16].

### Rule-based method [17-24]

This requires a database of reaction rules constructed from known metabolic reactions, and attempts to predict a metabolic pathway as a sequence of reaction rules. As a feature of reaction rules, some techniques focus on physicochemical properties and structures [25], while other methods focus on enzyme and gene information [26,27]. Since prediction ability depends on the size and type of metabolites used to build reaction rules, comprehensive prediction is difficult using the exhaustive search algorithm such as breadth-first search [23,24], and the approach has only been used to predict specific pathways and enzymes. In addition, the complexity of the features used to construct the reaction rules is a factor that has made comprehensive prediction difficult.

This study aims at a comprehensive and *de novo *approach to predict metabolic pathways between two arbitrary known or unknown compounds, and belongs to the rule-based methods. Using a simple feature that focuses only on the structural formula of compounds, our method enables comprehensive prediction that has been difficult for the conventional methods. Enzyme reactions on the metabolic pathways are used as the reaction rules, and are extracted from the KEGG database [28]. The feature on which we focus is the information before and after the structural change of the compound caused by the enzyme reaction. By using the information about this structural change, our method predicts the enzyme reactions that give the shortest path between two compounds for a given query. Further, a constraint for applying an enzyme reaction rule to a compound is set as the substrate inclusion condition, that is, the compound must include the substrate of the enzyme reaction as part of its own structure. This constraint and shortest-path strategy lead to *de novo *prediction of unknown biosynthetic pathways that a knowledge-based approach [17,18] cannot predict. In this study, the metabolic pathway prediction problem is reduced to the shortest path problem, and the search method is based on A* algorithm to traverse nodes in the order of priority and employs the LP solution as an admissible heuristics for estimating the distance to the goal.

## Methods

First, a chemical compound is represented by a feature vector which counts the frequencies of substructures in the structural formula. Second, a set of enzyme reaction rules is collected from the KEGG pathway database. Third, a reaction rule is represented as an operator vector by detecting the structural change to compounds before and after the reaction. Fourth, by defining compound vectors as nodes and operators as edges, prediction of a reaction pathway from a start compound to a goal compound is reduced to the shortest path search problem in the vector space. Then, "the output for reaction pathway prediction consists of a sequence of applied reaction rules". The A* algorithm is used to efficiently search for the shortest path. Finally, the Linear Programming (LP) algorithm is used as an admissible heuristic for estimating the distance to the goal.

### KEGG reaction data

The data for compounds and metabolic enzyme reaction information used in this method all come from KEGG. First, we extracted the information pathways from KEGG pathway [29]. By using the KEGG API, we concretely collected all enzyme reactions registered in the global map on the KEGG pathway. In the KEGG Reaction, a pair of compounds that are registered as "main" before and after the reaction indicate that it is a metabolic reaction present in KEGG enzyme or the KEGG pathway global map. In this study, the 2D-SDF structure was extracted only for those pairs registered as "main" to ensure the focus on compound metabolic reactions. Further, most KEGG reactions are registered as reversible reaction, and therefore, the forward and reverse directions were treated as a separate reaction. As a result, the 14570 enzyme reactions and the 6073 related compounds were obtained.

### Representation of chemical compounds and enzyme reactions

A key idea in our method is that a chemical compound is converted to a feature vector that represents substructure statistics extracted from the structural formula of the compound. This feature-vector representation evaluates whether a feature, such as a specific substructure, exists in a chemical compound or how many times that feature appears. This converts information about compounds into numerical vectors, called feature vectors, whose *i*th value corresponds to the existence or frequency of the *i*th feature considered. This feature-vector representation enables us to reduce the pathway search problem to a computationally feasible problem in the vector space, as will be discussed later in detail.

Substructures or paths extracted from chemical structures, which are regarded as graphs with atoms as nodes and bonds as edges, can be an effective descriptor of chemical compounds [30-32]. In this study, the feature vector based on the 2D chemical structure is defined as the number of appearances of each path in the structure of the chemical compound as follows:

(1) $$D_{l}^{u}\left(c\right)={\left(f_{c}\left(p\right)\right)}_{p\in P_{l}^{u},}$$

where $P_{l}^{u}$ is a set of paths whose length (depth), or number of bonds, is between *l *and *u *(*u *≥ *l*) and which appear at least once in the chemical structures in the dataset. *f_c_*(*p*) is the number of appearances of path *p *in the structure of a chemical compound *c*.

For example, methane (CH_4_),

(2) $$\begin{array}{ccc} & \text{H} & \\ & | & \\ \text{H-} & \text{C-} & \text{H}\\ & | & \\ & \text{H} & \end{array},$$

can be represented by the following feature vector:

(3) $$D_{0}^{2}({\text{CH}}_{4})=(\overset{\left(\text{C}\right)}{1},\overset{\left(\text{H}\right)}{4},\overset{\left(\text{O}\right)}{0},\overset{\left(\text{C-H}\right)}{4},...,\overset{\left(\text{O-H}\right)}{0},...,\overset{\left(\text{H-C-H}\right)}{6},...,\overset{\left(\text{O=C-H}\right)}{0}).$$

We call the path length range specified by *l *and *u *the "representation-depth", and denote it by "depth *l*-*u*", which is crucial for the expressiveness of vector representation.

**Figure 1:**
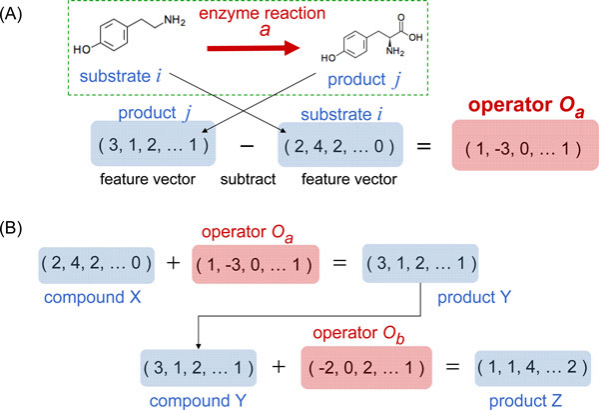
**Operator vector of enzyme reaction and a sequence of applications of operator vectors**. (A) An operator vector expresses the change in chemical structure before and after the reaction, which is computed as the difference between the product compound vector and the substrate compound vector. (B) An application of an enzyme reaction to a compound can be done simply by "addition" of the operator vector to the compound vector. Therefore, a reaction pathway is represented by a sequence of additions of operator vectors.

According to the feature-vector representation of chemical compounds, every enzyme reaction rule in the 14570 KEGG enzyme reactions is represented as an operator vector. An operator vector expresses the change in chemical structure before and after the reaction, which is computed as the subtraction of the substrate compound vector from the product compound vector: Let *i *denote the substrate compound of an enzyme reaction *a *and *j *denote the product compound of *a*. Let $D_{l}^{u}\left(i\right)$ and $D_{l}^{u}\left(j\right)$ denote the vector representation of *i *and *j*, respectively. Then, the operator vector *O_a _*for the reaction *a *is defined as (see also Figure 1):

$$O_{a}=D_{l}^{u}\left(j\right)-D_{l}^{u}\left(i\right).$$

Further, every reaction rule *R_a _*for the reaction *a *is defined as a pair *R_a _*= (*U_a_, O_a_*) of the substrate vector $U_{a}\left(=D_{l}^{u}\left(i\right)\right)$ and the operator vector *O_a_*. As a result, an application of the enzyme reaction to a compound can be achieved simply by "addition" of the operator vector to the compound vector (see also Figure 1), and a reaction pathway from a start compound *S *to a goal compound *G *is represented by a sequence of applications (additions) of operator vectors:

$S\overset{R_{1}=\left(U_{1},O_{1}\right)}{\underset{U_{1}\leq S}{\to }}X_{1}\left(=S+O_{1}\right)\overset{R_{2}=\left(U_{2},O_{2}\right)}{\underset{U_{2\;}\leq X_{1}}{\to }}X_{2}\left(=X_{1}+O_{2}\right)\to \cdots \to X_{n-1}\overset{R_{n}=\left(U_{n},O_{n}\right)}{\underset{U_{n}\leq X_{n-1}}{\to }}G.$

In this method, different reactions may sometimes be represented by the same vector because of insufficient short-length path counts in the compound vector.

#### Two constraint conditions for applying enzyme reaction rules

**Figure 2:**
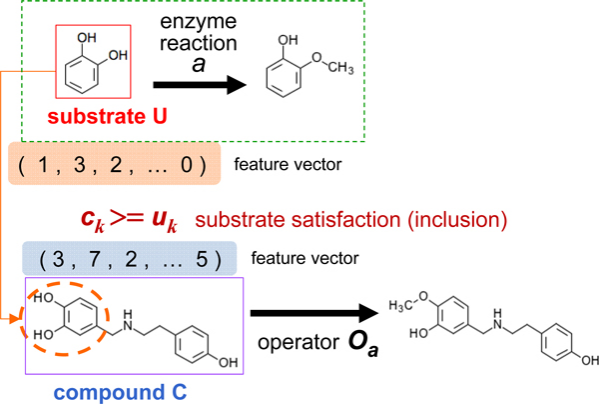
**Substrate satisfaction condition for applying enzyme reactions**. As a constraint for applying a reaction rule to a compound, the substrate inclusion condition is modeled by inclusion of the substrate vector. When attempting to apply the reaction rule *R *= (*U, O*) to a compound vector *C*, the compound vector *C *must include the substrate vector *U *as part of its own structure. That is, for any *k*, the value *c_k _*in the compound vector *C *= (*c*_1_, ..., *c_n_*) must exceed the value *u_k _*in the substrate vector *U *= (*u*_1_, ..., *u_n_*).

As a constraint for applying a reaction rule to a compound, the substrate inclusion condition is set as inclusion of the substrate vector. When attempting to apply the operator vector of a reaction rule *R *= (*U, O*) to a compound vector *C*, the compound vector *C *must include the substrate vector *U *as part of its own structure (see Figure 2). The precise determination procedure requires the entries *c_k _*in the compound vector *C *= (*c*_1_, ..., *c_n_*) to exceed the corresponding value *u_k _*in the substrate vector *U *= (*u*_1_, ..., *u_n_*), as represented by the following formula:

(4) $$\begin{array}{cc} c_{k}\geq u_{k}, & \left(1\leq k\leq n,C=\left(c_{1},\ldots ,c_{n}\right),U=\left(u_{1},\ldots ,u_{n}\right)\right). \end{array}$$

Note that this computationally easy procedure for substrate inclusion is a great advantage of our method using vector representation, because the graph inclusion problem for determining whether a compound structure contains a substrate structure is computationally hard (NP-hard).

The second constraint is the "non-negative" compound-vector condition. Since the operator vector *O *denotes a change in structure before and after the reaction, it sometimes contains negative values and the application (addition) of this to a compound vector *C *may produce a vector containing negative values. A compound vector is a vector that we define as representing the frequency of occurrence of partial structures, so negative values are not appropriate. Therefore, we set the following non-negative condition to filter out compound vectors containing negative values, preventing inappropriate products that contain negative values as a result of applying an operator vector.

(5) $$\begin{array}{cc} c_{k}+o_{k}\geq 0, & \left(1\leq k\leq n,C=\left(c_{1},\ldots ,c_{n}\right),O=\left(o_{1},\ldots ,o_{n}\right)\right). \end{array}$$

### Search algorithm between two compounds

**Figure 3:**
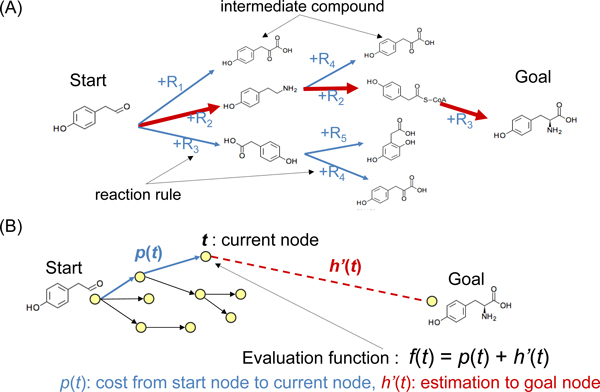
Search space of pathway prediction and A* algorithm for shortest path search. (A) The metabolic pathway prediction problem between compounds can be replaced by a mathematical shortest path search problem. That is, finding the shortest path to reach the integer vector of a goal compound by adding the integer vectors of reaction rules to the integer vectors of intermediate compounds can be considered a shortest path problem in an integer-vector space. (B) In the A* algorithm, the evaluation function (the distance-plus-cost heuristic) *f*(*t*) for each node *t *in the search space is defined as the sum of two functions: the past-cost function *p*(*t*), which is the cost (distance) it has taken to get from the starting node to the current node *t*, and a heuristic estimate *h*'(*t*) of the distance to the goal.

The purpose of this study is, given a start compound *S *and a goal compound *G*, to find the pathway leading to *G *by applying reaction rules. Here, the distance of a pathway between *S *and *G *is defined as "the number of rule applications (i.e., path length)". By introducing this distance definition, the metabolic pathway prediction problem between compounds can be replaced by a mathematical shortest path search problem. That is, finding the shortest path for reaching the integer vector of the goal compound by successively adding integer vectors of reaction rules to integer vectors of intermediate compounds can be considered as the problem of finding the shortest path in the integer-vector space. In this search space, a node is an intermediate compound vector and an edge is an applied reaction operator vector (see Figure 3).

### A* algorithm and heuristics

The A* algorithm uses a best-first search and finds a least-cost path from a given start node to a goal node. It uses a distance-plus-cost heuristic function to determine the order in which the search algorithm visits nodes to be explored in the search space. In the A* algorithm, the evaluation function (the distance-plus-cost heuristic) *f*(*t*) for each node *t *in the search space is defined as the sum of two functions, the past-cost function *p*(*t*), which is the cost (distance) it has taken to get from the start node to the current node *t*, and a heuristic estimate *h*'(*t*) of the distance to the goal (see Figure 3):

(6) $$f\left(t\right)\text{ = }p\left(t\right)\text{ + }h^{\prime }\left(t\right)\text{.}$$

In addition, the condition that ensures the A* algorithm finds a shortest path is expressed by the following formula:

(7) $$h^{\prime }\left(t\right)\;\leq h\left(t\right),$$

where *h*(*t*) is the true distance to the goal. That is, a heuristic function *h*'(*t*) that always underestimates the distance to the goal is required. Such a heuristic function *h*'(*t*) is referred to as an "admissible heuristics". If a given heuristic is admissible, the A* algorithm will reliably find a shortest path. The A* algorithm was implemented using the data structure "sorted priority queue" for maintaining the nodes to be traversed with weights of the evaluation function value *f*(*t*), while the breadth-first search uses the simple "queue" for the nodes to be traversed with no weight.

#### Breadth-first search (exhaustive search)

By setting the heuristic function *h*'(*t*) to zero for any node *t*, the A* algorithm becomes equivalent to the breadth-first (BF) search as exhaustive search.

#### Manhattan distance

Since each node *t *is an *n*-dimensional vector representing a compound, the most simple heuristic function *h*'(*t*) is to use the Manhattan (MH) distance, denoted by *MH*(*t, G*), between the current node vector *t *and the goal node vector *G*:

(8) $$MH\left(t,G\right)=\underset{i}{\sum }\begin{array}{cc} |t_{i}-g_{i}|, & \left(t=\left(t_{1},\ldots ,t_{n}\right),G=\left(g_{1},\ldots ,g_{n}\right)\right). \end{array}$$

However, naive use of the MH distance is inadmissible and does not guarantee the shortest path solution. Therefore, we use the following modified MH heuristic function *h*'(*t*):

(9) $$h^{\prime }\left(t\right)=\frac{MH\left(t,G\right)}{\left||O_{max}|\right|},$$

where ||*O_max_*|| represents the maximum norm among all of the operator vectors. The MH distance divided by this norm becomes an admissible heuristic, because this modified MH distance indicates the number of times the goal node *G *is reached by only applying the largest norm operator, and hence does not exceed the true distance to the goal node.

#### Linear programming (LP) heuristics

A path from the current node *t *to the goal node *G *is represented by a sequence of reaction rules *R*_1_, ..., *R_m_*, and hence the difference Δ*T *= *G *- *t *between the current node vector *t *= (*t*_1_, ..., *t_n_*) and the goal node vector *G *= (*g*_1_, ..., *g_n_*) can be represented by a linear sum of operator vectors $O_{k}=\left(o_{{}_{k}1},\ldots ,o_{{}_{k}n}\right)\left(R_{k}=\left(U_{k},O_{k}\right),k=1,\ldots ,m\right)$. Let *w_k _*denote the number of times that the operator vector *O_k _*is applied. Then the difference Δ*T *between the current node *t *and the goal node *G *may be expressed as follows:

(10) $$\Delta T=\underset{k}{\sum }w_{k}O_{k},$$

and the sum of the coefficients *w_k _*is exactly equal to the distance (the number of applications of operators) between the current node and the goal node. Now, consider the following optimization problem for the current node vector *t *= (*t*_1_, ..., *t_n_*) and the goal node vector *G *= (*g*_1_, ..., *g_n_*):

(11) $$\begin{array}{cc} \text{Minimize} & \underset{k}{\sum }w_{k} \end{array}$$

(12) $$\begin{array}{cc} \text{subject to} & G-t=\underset{k}{\sum }w_{k}O_{k}, \end{array}$$

(13) $$w_{k}\text{ is a non - negative integer}\text{.}$$

This optimization problem is an Integer Programming (IP) problem. The solution to this problem is similar to that for the shortest reaction path problem between the start node and the goal node, except that it does not take into account the order of application of the reaction rules and it ignores the constraint conditions when applying reaction rules. Nevertheless, the solution to "minimize ∑*_k _w_k_*" provides the tightest underbound for estimating the distance from the current node *t *to the goal node *G*, and it is obviously admissible. However, a critical defect is that the IP problem is computationally hard.

Our approach is to relax the constraints on the optimization problem "minimize ∑*_k _w_k_*" and to treat *w_k _*as a real number rather than an integer, that is, "continuous relaxation". The optimization problem now becomes an LP problem that can be solved in polynomial time. Note that allowing *w_k _*to be a real number means that we may apply an operator a real number of times, for example "apply the operator 1.5 times". In other words, a real-valued solution for the optimization problem "minimize ∑*_k _w_k_*" can be considered as the shortest distance to the goal node in a real vector space. In addition, it is well known and obvious that the real solutions for the optimization problem "minimize ∑*_k _w_k_*" with linear equation constraints are always smaller than the integer solutions. Therefore, the LP solution is admissible for guaranteeing the shortest path. We use this value as the LP heuristic function, which is another advantage of our method using the vector representation.

For solving the LP heuristic, we used IBM ILOG CPLEX in [33]. CPLEX is one of the fastest optimization problem solvers, and can be used for linear programming, quadratic programming, constraint programming, mixed integer programming, and is applicable to large-scale problems.

## Results

### Datasets and target pathways

#### KEGG Reaction dataset

Table 1 shows the number of operator vectors represented from the 14570 KEGG enzyme reaction dataset. There are three reasons that the total number of operators represented is less than 14570, the number of enzyme reactions registered in KEGG:

**Table 1:** Reaction rules for the whole KEGG pathway database

Representation-depth	0-1	0-2	0-3
Dimensionality of vector representation	76	254	653
Number of operator vectors *O*	4240	5542	8108

This table shows the relationship between the representation-depth of enzyme reactions and the number of unique reaction rules.

1. Some reactions are registered as different in KEGG, but the changes in structure are the same and only the substrates are different.

2. Some reactions are actually different but are represented by the same vector.

3. The structure registered as "main" is unchanged by the reaction.

The weakness of the second reason can be reduced by increasing the representation-depth for the vectors, which increases the number of reactions distinguished due to the improved expressive power.

#### DDT degradation pathway

In this study, we used the well-known DDT degradation pathway data set [34] as pathway data to verify the validity of our method. DDT is a chemical substance that can be synthesized for minimal cost, and began to be used as an insecticide during the 1940s because of its insecticidal action against many insects. However, the human carcinogenicity of DDT and its long-term persistency in the environment has since been pointed out [35]. It is important to evaluate the negative impact on the environment, and human health studies analyzing the metabolism of DDT has continued in recent years [36].

**Figure 4:**
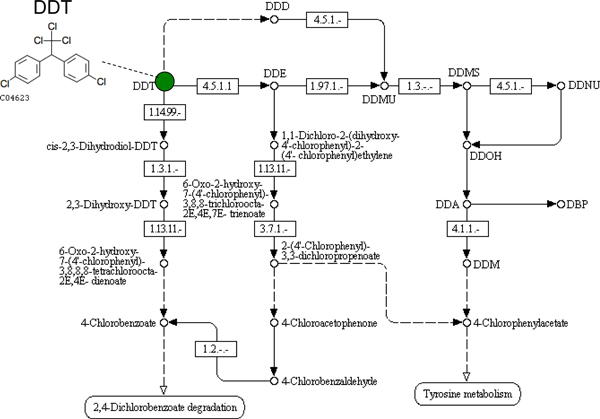
**DDT degradation pathway**. DDT stands for dichlorodiphenyltrichloroethane. DDT is a chemical that began to be used as an insecticide after showing insecticidal action against many insects in very small quantities. It is important to evaluate the negative impact on the environment, and human health studies on the metabolism of DDT have been done in recent years. This pathway consists of 20 compounds and 46 enzyme reactions.

Taking into account the number of involved pathways and compounds, as well as the fact that the pathway is a closed circuit, we consider the DDT degradation pathway ideal for verifying our approach. The pathway consists of 20 compounds and 46 enzyme reactions (Figure 4).

Table 2 shows the number of reactions represented as operator vectors associated with the 46 enzyme reactions from the DDT pathway.

**Table 2:** Reaction rules only for the DDT degradation pathway

Representation-depth	0-1	0-2	0-3
Number of operator vectors	38	44	46

This table shows the number of reaction rules focusing only on the DDT degradation pathway in the KEGG database.

In our experiments, 20 × 19 = 380 pathway routes were selected for the search problem. The first validation experiment only used the 46 enzyme reaction rules contained in the DDT degradation pathway. In the second "more general" experiment, all KEGG reaction rules were used to search the DDT pathway.

### Reconstruction of DDT pathway by shortest path finding

We first verified that the shortest path between the start node and the goal node implied the true metabolic pathway, identifying the shortest path using a BF search. Table 3 shows the percentage for which the true distance and the shortest distance are equal, and the rates at which the true pathway and the shortest-distance path match, for each representation-depth. The agreement rate with the true pathway increased as the representation-depth became larger, and the depth 0-3 gave an agreement rate of 100%.

**Table 3:** Agreement rate with the true pathway

depth	0-1	0-2	0-3
Agreement of the distance (%)	93.2	98.4	100
Agreement of the route (%)	81.3	93.2	100

This table shows the agreement percentage between the true distance and the shortest distance (points on *y *= *x*), and the rates at which the true pathway and the shortest-distance path match.

### Computational times for heuristics

Table 4 shows the computational time for searching for the shortest paths in the DDT pathway for each heuristic and each representation-depth.

**Table 4:** Average computational time (seconds/pair) for finding 380 pathway routes

depth	BF	MH	LP
0-1	1534	27.9	0.872
0-2	52.1	0.255	0.0325
0-3	0.0240	0.0314	0.0310

This table shows the computational time for each heuristic and each representation-depth to search for the shortest paths in the DDT pathway.

(BF: breadth-first search, MH: MH heuristic, LP: LP heuristic.)

Comparing the efficiency of the heuristic functions in this table showed in particular that a significant reduction in computational time was achieved by the LP heuristic. On the other hand, in the depth 0-3, reduction in computation time was not seen for most heuristics. This implies that, as the representation depth increases, the substrate inclusion condition works more effectively, and the number of branches in the search space becomes smaller.

Table 5 indicates the number of times that the search algorithm branched for each heuristic and each representation-depth. From Table 5, we observed a decrease in the search space with the improved heuristics at depth 0-1 and depth 0-2. A similar trend was observed at depth 0-3, but because the effect of the bound by the substrate inclusion condition was large for the more expressive representations at depth 0-3, the reduction in actual computation time was limited in contrast.

**Table 5:** Average number of branchings in the search (#branch/pair)

depth	BF	MH	LP
0-1	8389	1572	225
0-2	1575	129.4	17.4
0-3	12.8	11.6	7.6

This table indicates the number of times that the search algorithm branched for each heuristic and each representation-depth.

### Prediction of DDT pathway using all KEGG reaction rules

A more general reconstruction problem for DDT pathway was carried out using all KEGG reaction rules, to verify whether the method is practical for comprehensively reproducing the DDT degradation pathways. Table 6 shows the computational time for each heuristic and each representation-depth when all KEGG reaction rules were used to search for the shortest DDT pathway. Since increasing the number of operators increases the search space exponentially, none of the heuristics was able to accomplish the task within a realistic computational time (time out, or memory out) at depth 0-1 and depth 0-2. At depth 0-3, the BF search and the MH heuristics were unable to explore the solution space in a realistic computational time, and only the LP heuristic was able to calculate the shortest path solutions within a practical computational time.

**Table 6:** Average computational time (seconds/pair) using all KEGG reaction rules

depth	BF	MH	LP
0-1	N/A	N/A	N/A
0-2	N/A	N/A	N/A
0-3	N/A	N/A	61.9

This table shows the computational time for each heuristic and each representation-depth using all KEGG reaction rules to search for the shortest paths in the DDT pathway.

The agreement rate between the true distance and the true pathway route using the LP heuristic were 100% (380/380). Thus, despite using the generic operators (all KEGG reaction rules), the results showed that the method had high reproducibility.

### Prediction of Lutein biosynthesis pathway using all KEGG reaction rules

Another pathway prediction using all KEGG reaction rules was executed for Lutein biosynthesis pathway. Lutein biosynthesis pathway is a secondary metabolic pathway from the start compound "Lycopene" to the goal compound "Lutein". Lycopene is a red carotenoid and Lutein is a plant carotenoid, and there are two routes from Lycopene to Lutein in KEGG pathway database: the one is via Zeinoxanthin and the other is via *α*-Cryptoxanthin. The Lutein biosynthesis pathway has other difficulty compared with DDT pathway prediction: the structures of chemical compounds in the pathway are significantly larger than the ones in DDT pathway, and the KEGG pathway predictive tool PathPred [23,37] could not predict this pathway.

Our method with the LP heuristics succeeded to precisely predict all pathways between every pair of compounds on the Lutein biosynthesis pathway. The average computational time for the LP heuristic to predict the shortest paths for all pairs was 10.9 seconds. On the other hand, PathPred failed to predict the pathway between Lycopene and Lutein, where the default parameters of PathPred were used: "Simcomp Threshold" was set at 0.4, "Prediction cycle" was set at 1, and Reference pathway was set at "Biosynthesis of Secondary Metabolites (Plants)".

### Finding novel biochemical pathways for secondary metabolites of plant origin

To demonstrate the effectiveness of our method for finding novel pathways, we applied our method to predict a biochemical pathway for the start node "Delphinidin" and the goal node "Gentiodelphin". Both compounds are present in the KEGG database. Gentiodelphin is a plant-derived secondary metabolite associated with blue dye, and is known to be synthesized from Delphinidin [23]. The KEGG pathway predictive tool PathPred was also used for performance comparison.

**Figure 5:**
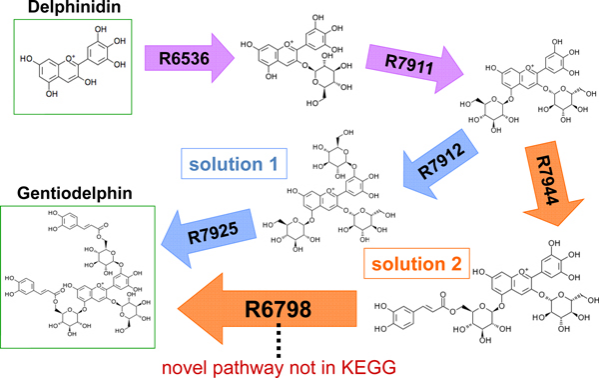
**Novel pathway finding for plant biochemical pathways**. "Gentiodelphin" is a plant-derived secondary metabolite associated with blue dye, and is known to be synthesized from "Delphinidin". The biochemical pathway was predicted with a start node of Delphinidin and a goal node of Gentiodelphin. The LP heuristic predicted the two shortest path solutions shown in this figure. The arrow indicates the reaction rule for routing information, accompanied by the KEGG reaction number. Both predicted pathways consist of four enzyme reactions. The first path (blue) is a metabolic pathway present in the KEGG pathway database. On the other hand, the second path (orange) is new and not registered in the KEGG database, and there is a possibility of a new route where the operator "R6798" is applied at the end.

Our method with the LP heuristics predicted the two shortest path solutions shown in Figure 5. Each arrow indicates the enzyme reaction as routing information, accompanied by the KEGG reaction number. Both predicted pathways consist of four enzyme reactions. The first path (blue) is a metabolic pathway present in the KEGG pathway database. The second path (orange) is new and not registered in the KEGG database, and there is the possibility of a new route where the enzyme reaction "R6798" is applied at the end. The computational times for the LP heuristic to predict the shortest paths were 35.0 seconds for the first solution, and 58.4 seconds for the second solution. PathPred only predicted the first pathway with a computational time of 1462 seconds, where the default parameters of PathPred were used.

Overall, our A*-based algorithm with the LP heuristic is more comprehensive and computationally efficient prediction method for biochemical pathway finding.

## Discussion

We have achieved high-speed pathway predictions using a vector-based search that simply focuses on the 2D structures of compounds. The A* algorithm guarantees the discovery of the shortest path, and the efficient search is achieved by the Linear Programming heuristic that estimates the distance to the goal. Results of verification experiments show the high reproducibility of KEGG pathways, the validity of the novel predicted pathway, and the versatility of our method.

### Search space for pathway predictions

An exponential increase in the search space accompanies an increase in the true distance. This is represented by the equation:

(14) $$P=N^{d},$$

where *P *is the size of the search space if all solutions are explored, *N *is the number of reaction rules, and *d *is the distance from the start node to the goal node. In the heuristic search, the search space can be reduced by visiting the nodes on the true path on a priority basis. Table 5 shows that the LP heuristic can significantly reduce the search space compared to searching all possible solutions.

In addition, taking into account the effect of the substrate inclusion condition that bounds the branching, the search space is improved as follows:

(15) $$P\;=\;{\left(N\left(\text{1 }-B\right)\right)}^{d},$$

where *B *is the ratio that bounds the branching, that is, the ratio at which operator applications are eliminated. When *B *increases, the base of the exponential function becomes smaller and hence the exponential increase can be reduced. That is, *B *plays a role in minimizing the exponential expansion of the search space. The significant reduction in computational time achieved by increasing the representation-depth for the vector representation is considered to be due to this reason. In other words, designing a high-speed searching method requires both an accurate heuristic function that estimates the distance to the goal and an effective bound on the branching to reduce the search space.

### Reproducibility of KEGG Pathway

Our experimental results for comprehensive predictions using all 8108 KEGG reaction rules show that our proposed method is able to reproduce enzyme reaction pathways in the KEGG pathway database with high accuracy. This is presumably due to the LP heuristic and bound on branching due to the substrate inclusion constraint on the vector representation.

### De novo prediction of known and unknown biosynthetic pathways

Our proposed method in this paper is a *de novo *prediction method in the sense that it does not require any initial network such as KEGG metabolic network as input and it is not a method just to traverse the pathway network. Our method takes as input the set of enzyme reaction rules collected from the KEGG pathway database. However, this does not necessarily imply that the pathway prediction using the list of all reaction rules is equal to the path search on KEGG pathway network. For each compound occurring at a node in KEGG pathway network, the KEGG network only contains the enzyme reactions whose substrate is exactly equal to the compound as an edge connected to the node. On the other hand, our method applies all reaction rules to a given compound if the compound is not only equal to the substrate of the reaction rule but also contains the substrate as a sub-structure (the substrate inclusion condition). Therefore, the search space of our method is exponentially larger than the KEGG pathway network. Further, our method is able to predict unknown biosynthetic pathways between two arbitrary known or unknown compounds.

## Conclusions

We have proposed a computationally efficient method to predict biochemical reaction pathways that derives a goal compound from a start compound. A chemical compound is represented by a feature vector that counts the frequencies of substructure occurrences in the structural formula. A set of enzyme reaction rules collected from the KEGG pathway database was represented using operator vectors, by determining the structural change in the compounds before and after the reaction. Two constraint conditions when applying reaction rules were substrate inclusion and compound formation. By defining each compound vector as a node and each operator as an edge, prediction of reaction pathways was reduced to the shortest path search problem in a vector space. We proposed an efficient search method that uses the A* algorithm for the shortest path search problem. We used an LP solution for heuristic estimation of the distance to the goal. The results showed that our method had high reproducibility for KEGG pathways and a high possibility of predicting new reaction pathways. We understand that we need larger-scale experiments to test the general performance and stability of our method on a number of various known pathways. This is one of our important future works. Also in the future work, the resulting shortest distance can be thought of as a kind of similarity measure between compounds that represents metabolic information, and hence applications to determining similarity of compounds for drug discovery such as [38-40] can be also expected.

## List of abbreviations

LP: Linear Programming; MH: Manhattan; BF: Breadth-first; IP: Integer Programming; DDT: dichlorodiphenyltrichloroethane.

## Competing interests

The authors declare that they have no competing interests.

## Authors' contributions

M.N. and Y.Sakakibara designed the study and analyzed the data. M.N. developed the system and performed the experiments. T.H., Y.Saito, and K.S. proposed the heuristics and analyzed the data. Y.Sakakibara wrote the manuscript. All authors read and approved the final manuscript.

## Author's information

Department of Biosciences and Informatics, Faculty of Science and Technology, Keio University, 3-14-1 Hiyoshi, Kohoku-ku, Yokohama 223-8522, Japan.
